# Internet of things based multi-sensor patient fall detection system

**DOI:** 10.1049/htl.2018.5121

**Published:** 2019-08-21

**Authors:** Sarah Khan, Ramsha Qamar, Rahma Zaheen, Abdul Rahman Al-Ali, Ahmad Al Nabulsi, Hasan Al-Nashash

**Affiliations:** 1 Department of Electrical Engineering, American University of Sharjah, Sharjah, United Arab Emirates; 2 Department of Computer Engineering, American University of Sharjah, Sharjah, United Arab Emirates

**Keywords:** pattern classification, body sensor networks, biomedical equipment, gyroscopes, geriatrics, Bayes methods, medical signal processing, microcomputers, accelerometers, patient monitoring, Internet of Things, nearest neighbour methods, cost-effective integrated system, credit card-sized single board microcomputer, visual-based classifier, sensor data, naive Bayes' classifiers, Internet of things based multisensor patient fall detection system, nonfall motions classification, k-nearest neighbour

## Abstract

Accidental falls of patients cannot be completely prevented. However, timely fall detection can help prevent further complications such as blood loss and unconsciousness. In this study, the authors present a cost-effective integrated system designed to remotely detect patient falls in hospitals in addition to classifying non-fall motions into activities of daily living. The proposed system is a wearable device that consists of a camera, gyroscope, and accelerometer that is interfaced with a credit card-sized single board microcomputer. The information received from the camera is used in a visual-based classifier and the sensor data is analysed using the *k*-Nearest Neighbour and Naïve Bayes' classifiers. Once a fall is detected, an attendant at the hospital is informed. Experimental results showed that the accuracy of the device in classifying fall versus non-fall activity is 95%. Other requirements and specifications are discussed in greater detail.

## 1 Introduction

The prime objective of this research project was to design an integrated system that not only detects patient falls in hospitals, but also accurately differentiates between different non-fall motions, which consist of activities of daily living (ADL). In this work, the ADL of hospital patients are limited to staying still in bed, lying down, sitting up, standing up, and bending. To be able to differentiate between a fall and non-fall motion, feature extraction, and various data classification methods were tested using signals and videos obtained for the activities mentioned.

According to the British newspaper ‘The Telegraph’, ∼208,720 falls occurred in National Health Service (NHS) hospitals in England by the end of October 2012, out of which 90 falls resulted in death and >50,000 patients were left injured [1]. The article confirms that 900 of the mentioned cases were classified as severe with patients suffering from hip fractures and brain injuries. Although it is almost impossible to prevent all falls in hospitals, the timely rescue of the patient can make a difference. Moreover, the extra cost of taking care of patients who have suffered a fall is an estimated £2.4 billion a year [1]. Accurate and timely fall detection can help save hospital's resources as well as help patients cope with the physical and emotional consequences of a fall.

Moreover, a 27-month fall prevalence study hosted in hospitals in the United States concluded that 315,817 accidental falls occurred in the duration of the study, out of which >78,000 resulted in injury and 600 resulted in death [2]. The study classified the likelihood of fall based on hospital units. For instance, neurology units have higher fall rates in hospitals, whereas patients in surgical and intensive care units have lower rates of fall. The study discovered that factors such as age, mental status, and illness severity were associated with the likelihood of fall in patients [2]. Similar to the article in The Telegraph mentioned earlier, this study also emphasises on how the length of hospital stay and costs related to fall injuries increase due to delay in rescue times, leaving the patient in emotional and physical trauma. However, an interesting fact mentioned in the study is that effective from October 2008, the federal agency within the US Department of Health and Human Service called Centers for Medicare and Medicaid Services (CMS), no longer pays for health care costs associated with falls during hospitalisation, since such events should never occur during hospitalisation [2]. Therefore, although accidental falls cannot be prevented completely at times, this paper presents the solution of a fall detection device for patients in hospitals that will accurately differentiate between fall and non-fall activities. The proposed system will help decrease costs associated with fall-related injuries for both the patient and the hospital and in turn, decrease prolonged hospital stays for patients through timely fall detection. Ensuring help arrives on time and any injuries sustained (hip fractures, brain injuries etc.) are treated immediately, can further prevent complications such as blood loss, bone infection, and pressure ulcers due to laying on the floor for prolonged hours etc.

There are products available in the market designed to detect falls for the elderly who live independently. However, the proposed device is a low-cost alternative designed for all ages and specifically for patients in hospitals. Moreover, the aim is to increase the accuracy of fall detection by classifying common actions performed by patients, such as sitting up and standing up. This was achieved by analysing the signals obtained from a combination of sensors whose inputs include tri-axial angular velocity, tri-axial acceleration, and performing data classification using methods such as the *k*-Nearest Neighbours (*k*-NN) and Naïve Bayes' classifiers. The system was integrated with a wearable camera and visual-based classification to further improve the accuracy of fall detection.

## 2 Related work

Upon reviewing the literature, several existing fall detection methods were found and compared with the proposed solution to perform trade-off analysis. The existing solutions are described below.

### 2.1 Three-dimensional (3D) accelerometer-based fall detection [3]

This system uses a 3D accelerometer as a sensor, a microcontroller, and a communication device. The device is placed at the waist and it is lightweight, uses a battery power supply and has low-energy consumption. It can detect falls as fall forward and fall backward, and non-fall motions such as standing or sitting, lying down with face up, face down, left lateral recumbent and right lateral recumbent. A 3D accelerometer is an electromechanical device that can measure dynamic acceleration and a static acceleration in three direction coordinates *x*, *y*, and *z*. The output from this device can be used to calculate the magnitude of acceleration, value of pitch (side-to-side motion) and roll (front-to-back motion) of a person and these are used to detect the mentioned positions and motions. Experimental results showed that the system can detect forward falls with an accuracy of 95% and backward falls with an accuracy of 75%. The advantage of this system is that it has low power consumption since there is only a single sensor being used for detection. However, the disadvantage is that it has limited accuracy and is prone to false detection because fast motions may sometimes be detected as a fall given that the solution only depends on acceleration quantities.

### 2.2 Smart Textile-based fall detection [4]

This system uses the smart textile by Hexoskin, Carré technology, which enables real-time remote monitoring of 3D acceleration data using three-axis sensors, cardiac activity, and respiratory activity on smartphones and tablets using Bluetooth. The system detects falls and classifies them into one of 11 categories: moving upstairs, moving downstairs, walking, running, standing, fall forward, fall backward, fall right, fall left, lying and sitting. Feature extraction is performed on the collected data using MATLAB and some of these features include amplitude, minimum, maximum, mean values, as well as the range and skew of the signal component. The system's main advantage is its flexibility and portability, since it can be used in any ambient environment without restrictions. Whereas, the main disadvantage is that it is a very expensive solution. Smart textile costs can often reach up to and over 3000 USD. Results of experiments carried out on 13 participants show the accuracy for fall detection to be 98% and fall orientation to be 98.5% with a system response time of ∼0.005 s.

### 2.3 Floor vibration-based fall detection [5]

This system consists of vibration-based floor detectors, using a piezoelectric sensor fixed to the floor surface of a room by means of a mass and spring arrangement. Combined with battery-powered processing electronics to evaluate the floor's vibration patterns, a fall signal is generated and a wireless transmitter sends the fall alarm to a communication gateway. The principle behind this system is that the vibrations on the floor generated by a human fall are significantly different from those generated by daily activities like walking and from those generated by objects falling on the floor. The main advantage is that the system is completely passive and unobtrusive to the person being monitored. However, the device has a limited range for detection, so if the patient were to move outside of this range, falls would not be detected. Experiments using a dummy showed that it has a high sensitivity of almost 100% while differentiating between a human fall and a falling object; however the difference between daily activities and human falls were not tested extensively.

### 2.4 Visual-based fall detection method [6]

This system uses vision-based approach and multivariate exponentially weighted moving average (MEWMA) scheme to detect a fall. The user is monitored through installed cameras and a fall or not fall decision is made based on four major steps of the algorithm. First, data acquisition is performed to record specific variables in order to determine whether the person has suffered a fall. Next is the segmentation phase which consists of extracting the body's silhouette from the input image sequence. In the third step, feature extraction is performed to extract discriminative human body attributes from the segmented frames obtained in the second step. The collected data is then used as input in fall detection and classification steps. Lastly, the presence and absence of a fall is determined in the detection phase. MEWMA is the classification technique used in this system to differentiate between a true fall and a false alarm. Experimental results showed that the system detects a fall with an accuracy of ∼90.5%. The advantage of this method is that the patient need not wear any device. However, a major drawback comes with the use of a surveillance camera, which makes this system unsuitable due to the intrusion of privacy. The patient may have the feeling of constantly being watched. Additionally, the range of fall detection is limited to visual coverage of the camera.

## 3 Method

The functional requirements (F.R.) are as follows:

*F.R. 1*: The system should be able to detect patient falls. Non-fall motions are classified into ADLs.
*F.R. 2*: The system should send a notification message when falls occurs.
*F.R. 3*: The system must have low power consumption.

The non-functional requirements (N.R.) are as follows:

*N.R. 1*: Wearable and wireless.
*N.R. 2*: Compact with dimensions 8 cm × 2 cm × 2 cm.
*N.R. 3*: Accuracy ≥95%.
*N.R. 4*: Low cost (under $150).
*N.R. 5*: Fast response time of utmost 15 s.

**Fig. 1:**
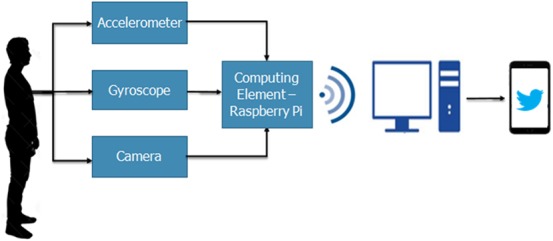
System block diagram

The wearable system hardware consists of three basic components, accelerometer, and gyroscope sensors, wearable camera and a single board microcomputer. The system hardware will henceforth be referred to as internet of things (IoT)-Multi-Sensor Unit. The technical specifications of these elements can be found in [7, 8, 9] along with the system block diagram depicted in Fig. 1.

**Fig. 2:**
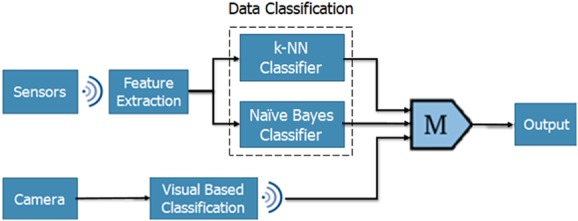
Classification system

As stated previously, the device differentiates between a fall and non-fall motion. If a fall is detected, the person-in-charge will be notified. This decision-making process requires the system to go through a series of steps that are represented in Fig. 2 shown below.

Sensor data and camera images are collected by the IoT-Multi-Sensor Unit. The sensor data is wirelessly transmitted to the PC, where feature extraction and data classification is performed on raw data in real time, using an IoT platform further explained in section 5. Moreover, to minimise power consumption of the device, the camera is made to operate in idle mode. It is switched on when the gyroscope data exceeds a certain pre-set threshold value, indicating a high risk of fall. The captured video is passed through a visual-based classifier on the microcomputer for image processing and the output is transferred to the IoT platform present on the PC.

Finally, the IoT platform categorises motion based on results from the classifiers. The output of the classifiers is binary where ‘logic 1’ indicates a fall. In the case of fall, a majority of the classifiers would output ‘logic 1’, shown using the majority gate in Fig. 2, and an automated Twitter notification would be sent to alert the person-in-charge. Feature extraction and data classification methods used to analyse data are explained in Sections 5.1 and 5.2.

In addition, one of the major advantages of the proposed system is that it allows the monitoring of multiple patients using a single PC. When data is transmitted by the microcomputer, it is identified by the PC using its IP address. Hence, the process of feature extraction and data classification for each patient is conducted in parallel.

To summarise, when power is supplied to the microcomputer, the accelerometer and gyroscope sense its inputs: angular velocity and acceleration in the *X-*, *Y-*, and *Z*-axis. Next, sensor data along with the result of the visual classifier is transmitted wirelessly to the PC. Feature extraction and data classification are performed on sensor data using the IoT platform. If a majority of the classifiers output ‘logic 1’, the final output of the system indicates a fall case and thus, a notification is sent out. Otherwise, the system will keep on detecting and classifying other motions.

## 4 Hardware architecture

The proposed system's hardware architecture consists of the IoT-Multi-Sensor Unit, wireless communication module, and a PC module.

### 4.1 IoT-Multi-Sensor Unit

This is an edge computing device, composed of a Raspberry Pi and sensor module. The Raspberry Pi is a credit card sized ARM-Microcomputer that has 1.4 MHz CPU speed, 1 GB flash memory, 32 GB SD memory card, an inter-integrated circuit communication port to interface the sensor module, a camera interface port, Wi-Fi access point, and an Ethernet port [7]. The sensor module comprises of two sensors, a three-axis accelerometer user programmable range and a three-axis gyroscope user programmable range [8] as well as a high-resolution image and video camera [9]. Table 1 shows a list of the Raspberry Pi peripherals used along with their current ratings. For instance, when the system is used as a stand-alone device, then only the gyroscope and accelerometer are operating, however, as mentioned previously, the camera only switches on once the sensor readings indicate a fall is likely to occur.

**Table 1:** Peripherals and current ratings (mA) [7–9]

Peripherals	Current rating, mA
gyroscope	36
accelerometer	0.5
camera	250
HDMI port	50
keyboard	100
mouse	100
wi-Fi	250
raspberry Pi-3 board	400
total consumption	1186.5

### 4.2 Communication module

It consists of Wi-Fi access points that link the IoT-Multi-Sensor unit and the IoT platform that can be accessed through the browser on a PC. Patients should be located within the Wi-Fi access point range of ∼50–60 m.

### 4.3 PC module

It is used to host the IoT platform known as ThingSpeak [10], which supports MATLAB and allows execution of programs in the cloud. The platform's functionality and relevant features are further detailed in the next section.

## 5 Software architecture

The software performs two main functions, namely feature extraction and data classification, implemented using ThingSpeak on the PC. The Python code on RPi extracts sensor readings and transmits them to ThingSpeak using RESTful application program interface (API), which is an API that uses HTTP requests to GET, PUT, POST, and DELETE data [11].

**Fig. 3:**
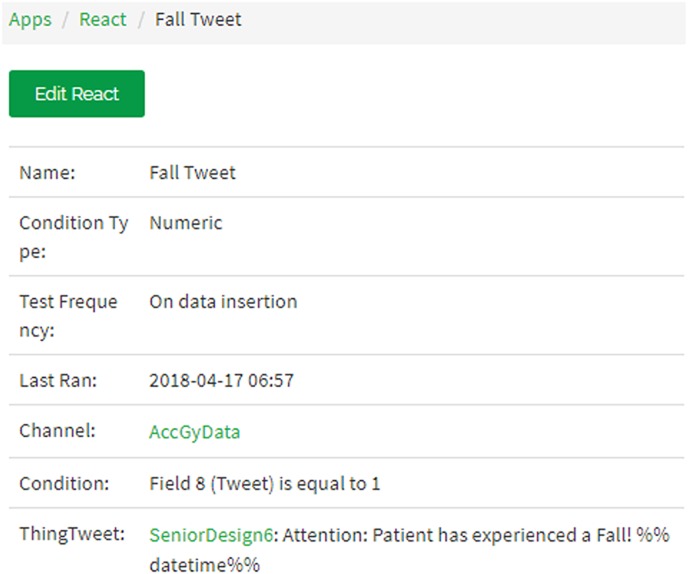
React App

ThingSpeak allows execution of MATLAB codes on an internet browser using its MATLAB Analytics app. The sensor readings along with the visual classifier result are imported to the MATLAB code to detect a fall. This platform also has the functionality of linking a Twitter account so that an automated Twitter notification may be triggered using its React app, which reacts to the results of the MATLAB code execution, as shown in Fig. 3. Thus, if the result of the data classifiers is a fall, a Twitter notification is sent to the care-taker and control room.

### 5.1 Feature extraction

**Fig. 4:**
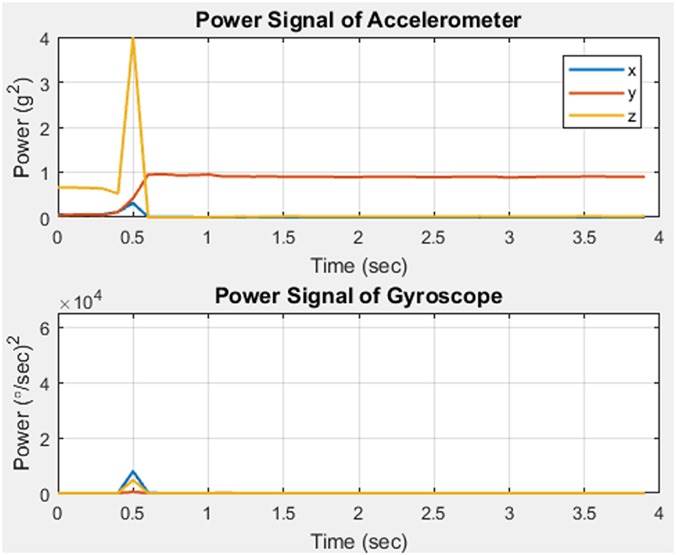
Fall case 1

**Fig. 5:**
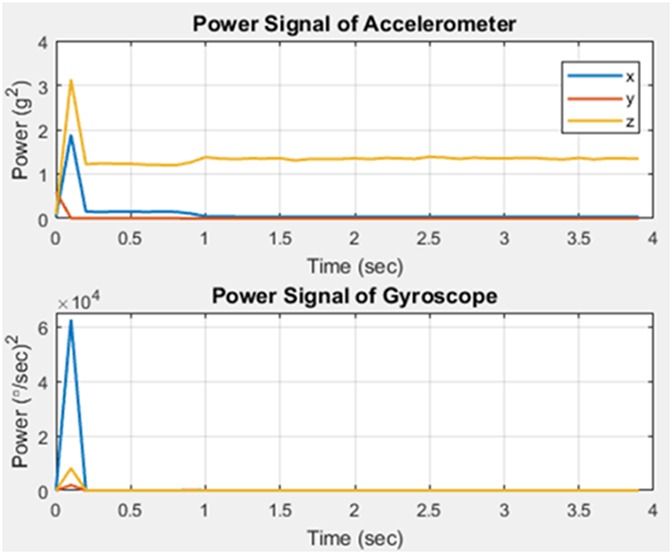
Fall case 2

In order to classify motions as fall or non-fall, patterns and regularities gathered from the data are evaluated. The pattern is analysed using feature extraction. This was carried out using the power signals of the acceleration and angular velocity in the *X*, *Y*, and *Z* directions. The graphs shown in Figs. 4 and 5 illustrate two different fall events.

Upon comparison of the two, it is observed that an increase in power at the fall instant for each axis differs according to the type of fall. Therefore, during a certain type of fall there could be a large increase in a single axis whilst having no significant change in the others. Using these results, it was concluded that acceleration and angular velocity in each of the three axes would have to be utilised in order to detect fall accurately. Therefore, the used features are as follows:

- Tri-dimensional acceleration signal power
- Tri-dimensional angular velocity signal power

### 5.2 Classifiers

Once the six features that are to be extracted from the sensor data were decided, they had to be utilised to classify whether an event is a fall or not. In order to choose a classifier, extensive research was carried out to determine the classifier with the highest accuracy; which was found to be the *k*-NN method. In an implementation of fall detection classification it was observed that there are several sophisticated classification algorithms available, however, for this research using *k*-NN classifier was sufficient due to the ease of implementation and relatively high accuracy [12]. To improve the fall detection accuracy, it was decided to use the Naïve Bayes' classifier as a second classifier working in parallel with the *k*-NN classifier.

Lastly, a visual-based classification method was also used in order to classify a motion as a fall or non-fall based on the visual information gathered by the wearable camera. Detailed explanations of the working of the three chosen classifiers are provided in the following sections.

#### 5.2.1 *k*-NN classifier

The *k*-NN classification method is a type of lazy learning, also known as instance-based learning. The lazy learning algorithm, instead of performing explicit generalisation, compares new testing data with data collected in training, which are stored in memory [13]. When predicting a class for new testing data, the distances or similarities between this test data and the training data is computed to make a decision [13]. To implement *k*-NN, the first task was to collect training data sets for all the possible classes, which in this case were still, lying down, sitting up, standing up, bending and fall. This was done by carrying out 40 different trials of all classes; however, having even larger training data sets would make the classifier more effective. As there were six features to be taken into account, the power of the accelerometer readings in the *X*, *Y*, and *Z* axes as well as the power of the gyroscope readings in the *X*, *Y*, and *Z* axes, the training and test data points exist in a 6D feature space.

The next step was to establish the value of *k*-NN. This is vital as the output of *k*-NN is the class that has the most frequent number of training data points amongst *k* of the nearest points around ‘*x*’. In order to determine which points are closest, the distances between ‘*x*’ and every training point have to be computed. There are several different measures of distance that can be used and some of these are the Euclidean distance, Hamming distance, and Manhattan distance [14]. Since all the six features are numerical values, the Euclidean measure is used where the distance between ‘*x*’ and a training point is given by the following expression [14], where *d* is six

(1) $$\sqrt {\mathop \sum \limits_{i = 1}^d {\left({x_i - y_i} \right)}^2} \eqno\lpar 1\rpar $$

Furthermore, the value of *k* has to be chosen appropriately because there are problems associated with values that are too large as well as values that are too small. Choosing *k* to be too small could lead to overfitting where the classifier becomes sensitive to outliers which could be the result of random fluctuations in the training data of a particular class [15]. This means that any outliers are picked up and learned as data belonging to that class [15]. Outliers do not characterise that particular class yet even if a few of those outliers happen to be nearest to the test point, the output will still be that class. However, if more nearest neighbours were considered, it would be clear that the test point actually belongs to a different class. On the other hand, if the value of *k* is too large, it leads to underfitting and defeats the basic theory behind *k*-NN, which is that training points that are nearest to the test point belong to the same class as the test point [15]. The optimal *k* value can be chosen through the process of cross-validation, which is a widely used method in machine learning. In this method the data is split into a number of folds so if there are *n* folds, then the first step of the algorithm is to use *n* − 1 of the folds as training data, and to use the single left-out fold as the test [16]. This is repeated *n* times until each fold has been used as the test set. A fixed value of *k* is used for every repetition and the error is calculated from this. The same process is repeated for different values of *k* and the one which gives the lowest error is the *k* that should be used for the *k*-NN classifier.

#### 5.2.2 Naïve Bayes’ classifier

The Naïve Bayes classifier is from a family of probabilistic classifiers which are able to predict, given an observation of an input, a probability distribution over a set of classes instead of only outputting the most likely class that the observation should belong to [17]. The Naïve Bayes classifier is based on applying Bayes' theorem of conditional probability and it assumes that the value of a particular feature is independent of the value of any other feature, given the class [18]. In this method of classification, the same training data sets for all classes that are used for *k*-NN is used again. Using these data sets, the mean and standard deviation of the classes is computed. Once these values are obtained the probability distribution of each of the test features, given every class can be computed (assuming they are a Gaussian distribution) using the following equation where ‘*x*’ is the value of the test feature ‘*F*_1_’ and ‘*C_k_*’ is the class [18]

(2) $$p\lpar F_1\; = \; x\vert C_k\rpar \; = \; \; \displaystyle{1 \over {\sigma \sqrt {2\pi } }}{\rm e}^{ - \lpar 1/2\rpar {\left({x - \mu /\sigma } \right)}^2}\eqno\lpar 2\rpar $$

The next step is to calculate the evidence, which is the total probability of all the features given each class. The evidence is given by the following expression where *k* is the total number of classes and *n* is the total number of features, which is six

(3) $$p\left({F_1\comma \; \ldots F_n} \right)\; = \; \mathop \sum \limits_k p\left({C_k} \right)p\lpar x\vert C_k\rpar \eqno\lpar 3\rpar $$

Once these values are acquired, the posterior probability of every class can be computed which is the conditional probability that is assigned after the relevant evidence is taken into account. In this context, ‘posterior’ means after taking into account the relevant evidence related to the particular case being examined [19]. The posterior probability of a class is given by the following equation

(4) $$p\lpar C_k\vert F\rpar \; = \displaystyle{{\,p\left({C_k} \right)p\lpar F_1\comma \; \ldots F_n\vert C_k\rpar } \over {\,p\left({F_1\comma \; \ldots F_n} \right)}}\eqno\lpar 4\rpar $$

Finally, after the posterior probability of each class is computed, the result of the Naïve Bayes' classifier is the class with the higher posterior probability.

#### 5.2.3 Visual-based classifier

A visual-based classifier is used to analyse the video recorded by the camera module. As expected, during a fall motion the camera will record abrupt changes in the external environment, whereas, a gradual change in the surroundings will be seen by the camera when a patient is carrying out ADLs. Therefore, the classifier measures the quantitative difference between frames rather than analysing each frame individually. The recorded video is broken down into frames and the classifier calculates the percentage difference between consecutive frames. If the calculated difference exceeds the threshold value, the motion is categorised as a fall. Through several trials, the threshold value for a fall motion was found to be 7%.

An easy way to comply with the requirements stated in the Author Guide [1] is to use this document as a template and simply type your text into it. PDF files are also accepted, so long as they follow the same style.

## 6 Implementation

**Fig. 6:**
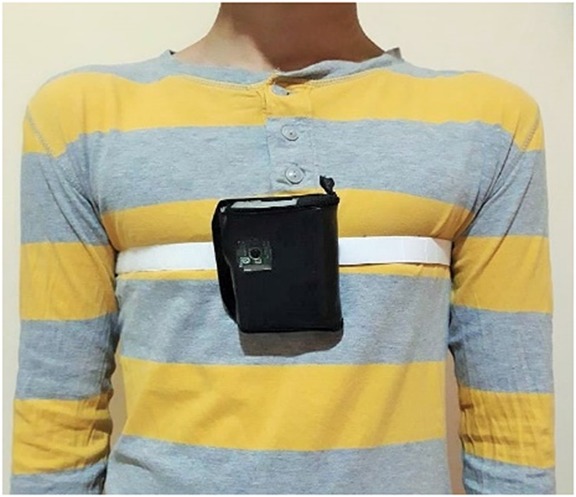
Wearable Multi-Sensor unit attached to the subject's chest

To validate the proposed hardware design and software algorithms, an experimental setup, shown in Fig. 6 was developed. The device is placed in a leather casing, which is attached to a Velcro strap. As shown in the figure, the wearable unit is strapped to the body such that the device is placed over the subject's chest. The strap ensures that the sensors are firmly attached to the body to prevent inaccurate readings, and thus false alarms.

The device is autonomous once switched on and the codes in Python and MATLAB are executed. A power bank was used to power the device, taking into account the total consumption of the peripherals mentioned in Table 1.

## 7 Results

**Fig. 7:**
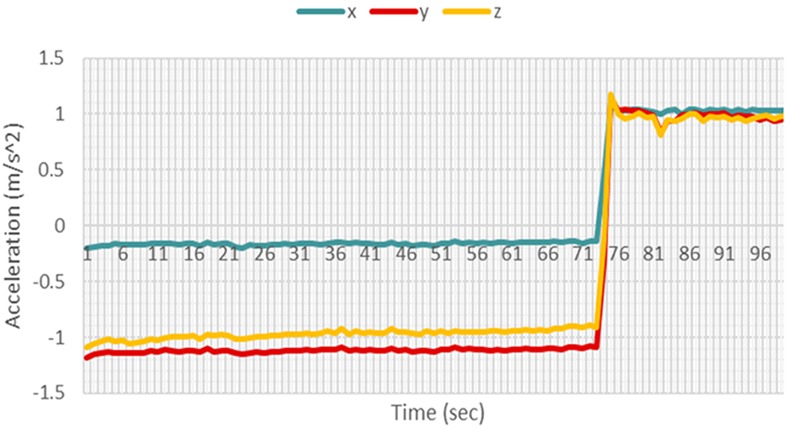
Accelerometer for a sit up motion

**Fig. 8:**
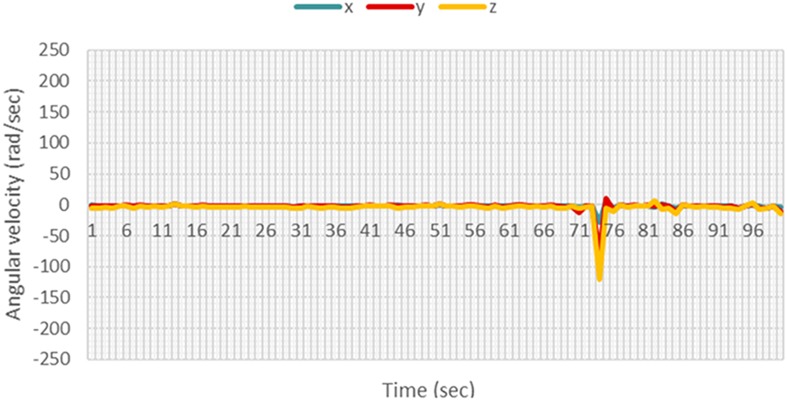
Gyroscope readings for a sit up motion

Test data were obtained using the same methods used for collecting training data. After carrying out the five non-fall motions many times, it was observed that the gyroscope and accelerometer readings in any axis do not exceed values larger than ±120 rad/s and ±1.5 m/s^2^, respectively, as shown in Figs. 7 and 8.

Hence, these values were chosen as the threshold at which the camera starts recording.

**Fig. 9:**
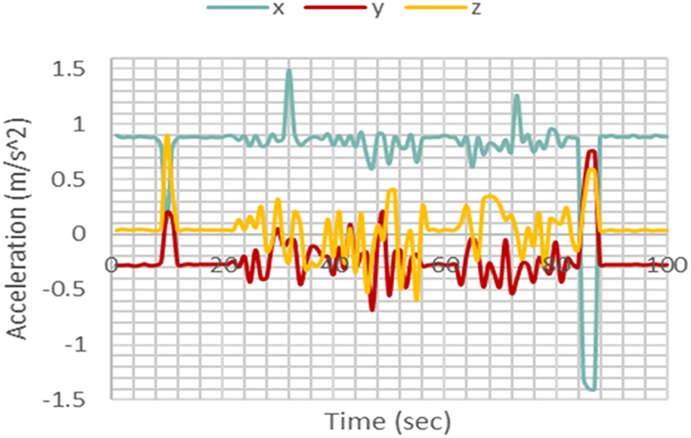
Accelerometer readings for a fall motion

**Fig. 10:**
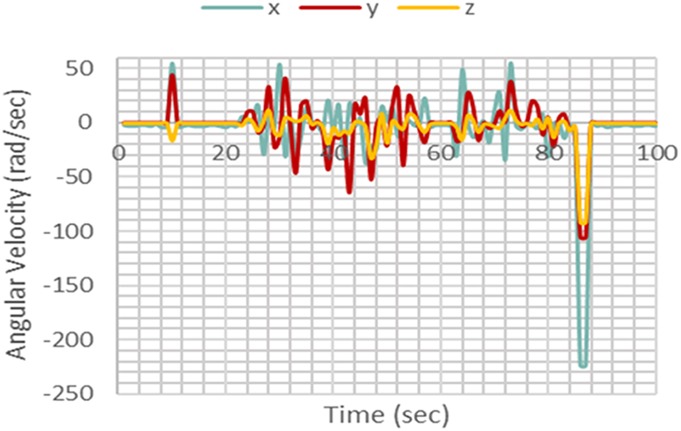
Gyroscope readings for a fall motion

When a fall motion occurs as illustrated in Figs. 9 and 10, the time of fall was apparent, as there was a significant increase in the sensor readings. For the specific case shown in the figures, though the accelerometer readings did not exceed the threshold of ±1.5 m/s^2^, the gyroscope readings exceeded the threshold of ±120 rad/s by a large margin. This significant increase allows the *k*NN and Naïve Bayes' classifiers to differentiate the fall from other non-fall motions.

**Fig. 11:**
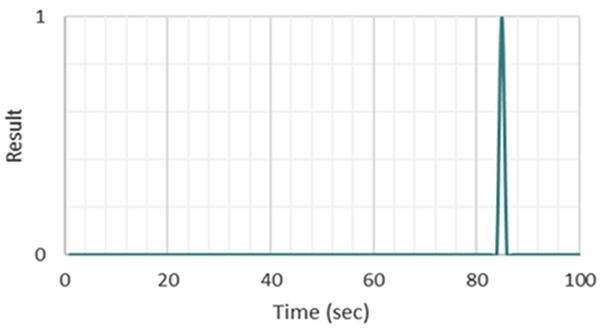
Visual-based classifier result

**Fig. 12:**
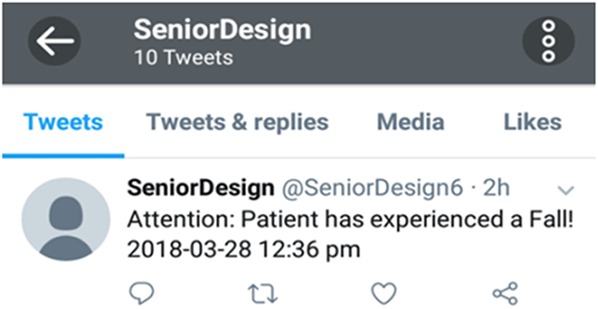
Twitter notification

Exceeding the threshold values implies that the camera records a video and a fall results in larger differences between frames in this video. When this difference surpasses 7%, the visual-based classifier gives a binary output representing a fall as shown in Fig. 11. When the system detects a fall, an automatic Tweet, shown in Fig. 12, is sent out to notify an attendant or caretaker.

To determine the accuracy of the system, an experiment was conducted while the wearable IoT unit is attached to the subject. Ten male and female human subjects aged 20–24 years old participated in the experiment. The subjects were instructed to perform six different common actions (movements) including standing still, laying down, sitting up, standing up, bending and falling. Falling was performed from still, sitting up, standing up, and bending positions. Hundred trails were executed by each subject. The outcomes of which are shown in the table below. The expected result for all the non-fall motions was negative, while the expected result for a fall case was positive. In the non-fall scenario, a true negative (TN) indicates correct detection of the absence of a fall, whereas a false positive (FP) indicates a false alarm. For fall case, a true positive (TP) indicates correct detection of a fall, whereas false negative (FN) indicates incorrect detection.

TP, TN, FP, and FN were used to calculate the accuracy, sensitivity, and specificity of the system. Sensitivity is the ability to detect a correct result when the activity (fall) is present and specificity is the ability to detect a correct result when the activity is absent [20]. The used formulae are as follows (Table 2)

(5) $${\rm Sensitivity\; } = {\rm \; \; }\displaystyle{{{\rm TP}} \over {{\rm TP + FN}}}\; 100\percnt \eqno\lpar 5\rpar $$

(6) $${\rm Specificity\; } = \; \; \displaystyle{{{\rm TN}} \over {{\rm TN + FP}}}\; 100\percnt \eqno\lpar 6\rpar $$

(7) $${\rm Accuracy\; } = {\rm \; \; }\displaystyle{{{\rm TN + TP}} \over {{\rm TN + TP + FN + FP}}}\; 100\percnt \eqno\lpar 7\rpar $$

**Table 2:** Trial outcomes

Position	Trials	Expected results	TP	TN	FP	FN
null (still)	100	negative	—	100	0	—
lying down	100	negative	—	97	3	—
sitting up	100	negative	—	96	4	—
standing up	100	negative	—	93	7	—
bending	100	negative	—	86	14	—
fall (still)	100	positive	100	—	—	0
fall (lying down)	100	positive	98	—	—	2
fall (sitting up)	100	positive	100	—	—	0
fall (standing up)	100	positive	93	—	—	7
fall (bending)	100	positive	90	—	—	10
total	1000	—	481	472	28	19

According to the formulas above, sensitivity, specificity, and accuracy were calculated to be 96.2%, 94.4%, and 95.3%, respectively.

## 8 Limitations

ThingSpeak limits the update rate of data to one reading/second, whereas the motion sensing chip collects and transmits data at a rate of ten readings/second. This difference in data rates at the transmitting and receiving ends may result in loss of readings and therefore, reduced overall system accuracy. Furthermore, the MATLAB compute time in ThingSpeak is restricted to 20 s; hence it does not allow the use of an infinite loop in the code, making it necessary to manually run the program every time a new reading is received. To resolve this issue, a Macro recorder and auto clicker was installed on the browser to run the program continuously.

## 9 Conclusions and recommendations

A compact, cost-effective and wearable wireless fall detection system was designed and developed. Patient data is transmitted in real time to the hospital server, thus enabling caretakers to remotely monitor patient status and receive twitter notifications when falls occur. It also permits monitoring of multiple patients simultaneously using the unique IP address of each device, making the system scalable and distributive. Moreover, the system is stand-alone, therefore its application is not only limited to hospital beds. Elderly who live alone can also use it in where notifications will be sent to their family members.

In order to enhance the accuracy of the system, the classifiers can be optimised by including classes for various other non-fall actions. Additionally, the overall size of the device can be reduced by replacing the power bank with rechargeable lithium batteries and hence increasing the patient's comfort level. The size of the device can be further reduced by a factor of two and the weight by a factor of five using the Raspberry Pi Zero instead of Raspberry Pi 3 Model B [21].
